# Serum anti-hepatitis B surface antigen in hemodialysis patients

**Published:** 2012-01-04

**Authors:** Mahmoud Rafieian-Kopaei, Hamid Nasri

**Affiliations:** ^1^Medical Plants Research Center, Shahrekord University of Medical Sciences, Shahrekord, Iran; ^2^Department of Nephrology, Division of Nephropathology, Isfahan University of Medical Sciences, Isfahan, Iran

**Keywords:** Hepatitis B virus, Hepatitis B virus, Hemodialysis, Anti-HBs antibody

## Abstract

To evaluate the immune response to hepatitis B vaccination in stable hemodialysis (HD) patients, a retro-prospective investigation was conducted on 68 HD patients. Participants were vaccinated against hepatitis B virus with an intramuscular hepatitis B vaccination schedule, 40 micrograms at 0, 1, and 6 months. The serum antibody level against hepatitis B surface antigen (HBs) in HD patients was 35±55. In this study, no significant differences of Anti-HBs antibody between diabetic and non-diabetics or male and female subjects were observed. There were not any significant correlation between antibody against HBs-Ag and serum albumin. There was not significant correlation between anti-HBs antibody and age, proportion of HD, duration of HD or dialysis efficacy. In this study, there was not significant correlation between serum antibody level against hepatitis B surface antigen and some demographic indices of HD patients, however, these findings need to re-test in other centers with more participants.

**Implication for health policy/practice/research/medical education:**

In a study on 68 hemodialysis participants, no significant differences of Anti-HBs antibody between diabetic and non-diabetics or male and female subjects were observed. There were not significant correlation between anti-HBs antibody and age, proportion of hemodialysis, duration of hemodialysis or hemodialysis efficacy. In this study, there was not significant correlation between serum antibody level against hepatitis B surface antigen and some demographic indices of hemodialysis patients, however, these findings need to re-test in other centers with more participants.

## Introduction

One of the reasons for increased mortality and morbidity in hemodialysis patients is due to high vulnerability to various infections like hepatitis B virus (HBV) (1). Vaccination against HBV vaccination is routinely done for all patients undergoing hemodialysis (HD) (1,2). Nevertheless, antibody production is much lower than in healthy persons (2,3). It has been observed that around 30–40% of HD patients fail to make antibody against HBV after vaccination (3,4). Also HD patients also have an incapability to keep appropriate antibody level during time (5). In this study, we aimed to examine some demographic indices which might influence the antibody production during HBV vaccination in a group of stable HD patients.

## Patients and Method

### Patients

This cross-sectional study was conducted on a group of HD patients under regular hemodialysis. Exclusion criteria included: using any antibiotics or NSAIDs, or existence of infection.

### Hepatitis B Vaccination

The vaccination of HD patients was started before the study through a schedule of 0, 1, and 6 months. They received 2 ml (40μg) of Euvax Hbs-Ag, through intramuscular deltoid injections.

### Laboratory Methods

Blood samples were collected for assessment of antibody to hepatitis B surface antigen (anti-HBs) by ELISA technique with Dialab kits (manufactured in Austria) at least 6 months after completion of initial vaccination series to assess response to vaccination. A participant had responded to the vaccine if the anti-HBS level was>10 mIU/ml. Subjects with levels 10–100 mIU/ml were assumed to be ‘poor responders’, while persons with levels>100 mIU/ml were considered as ‘good responders’ (1). To assess the efficacy of HD, urea reduction rate (URR) was calculated from pre- and post-blood urea nitrogen (BUN) data (6). Duration and proportion of HD sessions were determined from the patients’ records. The length of each HD session was 4 hours.

### Ethical issues

The research followed the tenets of the declaration of Helsinki; written informed consent was obtained and the research was approved by ethical committee of Shahrekord University of Medical Sciences.

### Statistical analysis

Data were determined as the mean±standard deviation (SD), median and range values. Comparison between the groups was performed using Student’s *t*-test. Statistical correlations were evaluated using partial correlation test. All statistical analyses were performed using SPSS (version 11.5). Statistical significance was determined at p< 0.05.

## Results

Total patients were 68 (female=19, male=49). The mean age of the patients was 53±18 years. The mean duration of HD was 27±29 months (Median: 22.8 months). The mean anti-HBS-Ab titer was 35±55 (median=5.5). There was not any significant difference of antibody production against HBS-Ag between male and female or diabetic and non-diabetic subjects (p= N.S). There was not significant association of serum albumin with antibody against anti-HBS-Ab. Also, there was not any significant association of anti-HBS-Ab titer with age, proportion of HD, duration of HD or URR, (p= N.S.).

## Discussion

In this study, there were not any significant differences of antibody production against hepatitis B surface antigen between diabetic and non-diabetics or male and female subjects. There was not significant association between anti-HBs antibody titer and age, proportion of hemodialysis, duration of hemodialysis or dialysis efficacy. While vaccination can offer significant protection against hepatitis B virus infection, vaccine response rates are low and unpredictable between dialysis patients (2–5). It was observed that 34–88% of vaccinated HD patients produced protective levels of anti-HB antibody, and few data exist assessing the persistence of this response (2,5). Because of the impaired immune response, HD patients are given larger doses of the vaccine and frequently require revaccination or booster doses to produce and maintain adequate anti-HB titers (7,8). Given the limited efficacy of hepatitis B vaccine in HD patients, it would be useful to define factors that may affect vaccine response in HD patients. In fact, unresponsiveness to HBV vaccine is multifactorial, and linked to the presence of several interacting factors. Various findings have shown that response rates are greatest among HD patients younger than 40 years (5) and sex may also influence vaccine response (8). However, other studies, the same as our findings have failed to show that sex or proportion of HD influence response to hepatitis B vaccine (3–5). Ibrahim *et al*. studied the evolution of anti-HBS Ag antibody after primary vaccination in 29 HD patients and showed no significant correlation with age and duration of HD therapy. They found that responders to primary vaccination had significantly higher levels of URR. They also found the response to hepatitis B vaccination in HD patients neither correlated with nutritional status nor age or systemic inflammation. However, adequate HD was correlated with good response to hepatitis B vaccine (9). Kara *et al*. in a retrospective study on 34 HD patients found that the immune response of the HBV vaccine was low in these patients and it was affected by some factors such as nutritional status (10). Kovacic *et al*. conducted a study to find out how dialysis adequacy affects response to HBV vaccination. They found that the group of good responders had a significantly better HD adequacy than the group of non-responders. Kt/V values showed a significantly positive correlation with the HBs-Ab level. They concluded that efficient HD significantly improved the response to vaccination against hepatitis B (11). Likewise Ocak *et al*. aimed to investigate whether HD patients suffering from diabetes mellitus could be considered at risk for the development of the protective antibodies to hepatitis B vaccination. They conducted a study on forty-nine HD patients and showed that diabetic patients on HD might carry a greater risk of not seroconverting than non-diabetic ones for antibody response to HB vaccination (12). Sorkhi *et al*. in a study on 62 HD patients showed duration of HD had no influence on response to vaccination (13).

## Conclusion

In this study, we concluded that factors associated with serum antibody level against hepatitis B surface antigen were different in our HD patients and various factors might be responsible for antibody production and more multicenter investigations need to define these related factors.

## Authors’ contributions

HN and MRK wrote the manuscript equally.

## Conflict of interests

The authors declared no competing interests.

## Ethical considerations

Ethical issues (including plagiarism, data fabrication, double publication) have been completely considered by the authors.

## Funding/Support

This study was supported by a grant from Shahrekord University of Medical Sciences, Iran. This paper was derived from M.D thesis.
